# Frog intestinal perfusion to evaluate drug permeability: application to p-gp and cyp3a4 substrates

**DOI:** 10.3389/fphar.2015.00141

**Published:** 2015-07-14

**Authors:** Neelima Yerasi, Himabindu Vurimindi, Krishna Devarakonda

**Affiliations:** ^1^Institute of Science and Technology, Jawaharlal Nehru Technological University, HyderabadIndia; ^2^Department of Pharmacology, University College of Pharmaceutical Sciences, Kakatiya University, WarangalIndia

**Keywords:** effective permeability coefficient, single pass intestinal perfusion, *P*-glycoprotein, CYP3A4, frog intestinal perfusion, *in situ* models for drug permeability assessment, P-gp CYP3A interaction studies

## Abstract

To evaluate the reliability of using *in situ* frog intestinal perfusion technique for permeability assessment of carrier transported drugs which are also substrates for CYP enzymes. Single Pass Intestinal Perfusion (SPIP) studies were performed in frogs of the species *Rana tigrina* using established method for rats with some modifications after inducing anesthesia. Effective permeability coefficient (*P*_eff_) of losartan and midazolam was calculated in the presence and absence of inhibitors using the parallel-tube model. P_eff_ of losartan when perfused alone was found to be 0.427 ± 0.27 × 10^-4^cm/s and when it was co-perfused with inhibitors, significant change in *P*_eff_ was observed. P_eff_ of midazolam when perfused alone was found to be 2.03 ± 0.07 × 10^-4^cm/s and when it was co-perfused with inhibitors, no significant change in *P*_eff_ was observed. Comparison of *P*_eff_ calculated in frog with that of other available models and also humans suggested that the *P*_eff_-values are comparable and reflected well with human intestinal permeability. It is possible to determine the *P*_eff_-value for compounds which are dual substrates of *P*-glycoprotein and CYP3A4 using *in situ* frog intestinal perfusion technique. The calculated *P*_eff_-values correlated well with reported *P*_eff_-values of probe drugs. comparison of the *P*_eff_-value of losartan obtained with that of reported human’s *P*_eff_ and Caco 2 cell data, and comparison of the *P*_eff_-value of midazolam with that of reported rat’s *P*_eff_, we could conclude that SPIP from model can be reliably used in preclinical studies for permeability estimation. This model may represent a valuable alternative to the low speed and high cost of conventional animal models (typically rodents) for the assessment of intestinal permeability.

## Introduction

In order to bring safe and effective drugs to the market it is crucial to understand the factors influencing drug absorption and predict these values on the basis of pre-clinical *in vitro* and/or *in situ* experiments (Lipka and Amidon, 1999). Drug absorption across the intestinal membrane is a culmination of complex processes. Passive absorption takes place by transcellular route (through the cell membrane of enterocytes) or by Para cellular route (through the tight junctions between the enterocytes). Active absorption occurs by carrier mediated transport or facilitated diffusion. “Various efflux transporters such as P-gp, BCRP, MRP2 are also present that might pump the drug back, thus limiting the absorption. Intestinal enzymes present metabolize drugs to alternate moieties” (Kim et al., 1995). Multivariate processes are involved in intestinal absorption of drugs. Hence it is difficult to use a single model to accurately predict the *in vivo* permeability characteristics of drug candidates.

Metabolism by cytochrome P4503A4, the major isoform of CYP3A subfamily, and mdr1 P-glycoprotein (P-gp), an ATP-binding cassette transmembrane transporter (ABC transporter) mediated efflux act as two important rate limiting biological barriers to drug absorption from the intestine. It is well documented that the metabolism/active efflux in the small intestine is involved in the poor bioavailability of many drugs (Krishna and Koltz, 1994; Cécile et al., 2007). CYP3A4 is mainly expressed in liver, but intestinal enterocytes also express considerable amounts of CYP3A4, substantial enough to alter bioavailability of many marketed drugs (Paine et al., 1997; Von Richter et al., 2004). P-glycoprotein is also expressed on the brush border membrane of enterocytes. The substrate specificity of CYP3A and P-gp overlap each other. As a result these two proteins act synergistically in reducing the bioavailability of their substrates after oral administration (Thummel et al., 1997; Ambudkar et al., 1999). Many drug–drug or drug–food interactions in preclinical and clinical studies have been associated with transporter mediated efflux (Varma et al., 2006). Hence it is essential to screen molecules for P-gp involvement during preclinical studies.

Several *in vitro, in vivo*, and *in situ* techniques have been reported for permeability studies involving P-gp and also CYP3A. *In vitro* models include cell lines which over-express P-gp (MDCK, Caco-2) either cDNA transfectants over expressing P-gp or non-transfected cell lines and also the *ex vivo* Using Chamber model using excised rat intestinal Segments (Tukker, 2000).

Though *in vitro* techniques have the advantage of generating large volumes of data, they are not thoroughly standardized and are associated with several limitations, hence less predictive. The Caco-2 cell model is routinely used to investigate drug transport because of its structural and physiological similarity to the intestinal epithelium, including the expression of P-gp (Balimane and Chong, 2005). However, quiescent Caco- 2 cells do not normally express CYP3A4 and also they do not always express appropriate amounts of transporters or enzymes (Artursson and Karlsson, 1991).

*In situ* single pass intestinal perfusion (SPIP) technique using different animal species including rat, rabbit, pig, dog, and monkey has been reported in literature to study the intestinal absorption of drugs. Among these animal models, SPIP in rat is a well-established technique to study the intestinal passive absorption of drugs with good correlation between human and rat intestinal absorption but for drugs whose intestinal permeability is driven by carrier-mediated absorption this is not the case. “Expression profiles of transporters and metabolizing enzymes in both rat and human intestines (duodenum and colon) were measured using Gene Chip analysis. There was no correlation between rat and humans (*r*^2^ = 0.29) in oral drug bioavailability, which also agreed with previous results” (Cao et al., 2006). Hence there is a clear need for the development of a novel model for the study of intestinal absorption of drugs involving transporters such as P-gp and CYP3A enzymes.

In this study we have developed SPIP using frog as the animal model for the assessment of intestinal permeability of drugs, which are substrates of P-gp, and CYP3A4 in humans. Frog small intestine has the same composition and villi as vertebrate membranes and same transport mechanisms and luminal enzymes have evolved from lower vertebrates to humans. This is further supported by a study, in which *in vitro* permeability coefficient deduced from isolated frog intestinal sac showed to be a reasonable predictor of *in vivo* oral absorption in humans for compounds that are passively absorbed (Trapani et al., 2004). Another study also indicated the expression of specific transporter systems in frog intestine (Franco et al., 2008). When compared with *in vitro* methods, SPIP provides an advantage of experimental control (e.g., permeate concentration, intestinal perfusion rate), intact intestinal blood supply, and barrier function of the intestine is not lost or compromised during the entire length of the experiment (Lennernäs, 2007). Tissue viability is much longer when compared with *in vitro* isolated intestinal segment models. In a study, we have demonstrated that the SPIP frog model can be used for the biopharmaceutical classification (Yerasi et al., 2015).

The objective of the present study was to evaluate the reliability of using *in situ* frog intestinal perfusion technique for permeability assessment of carrier transported drugs, which are also substrates for CYP3A4 enzymes. We have chosen the antihypertensive drug losartan as one of the probe drugs since it exhibits highly variable and low oral bioavailability (Lo et al., 1995). Losartan is a CYP3A4 substrate, which undergoes phase-1 metabolism in the intestine and active secretion in to the lumen by P-gp after absorption (Benet et al., 1996; Borst et al., 1997). The second probe drug is midazolam, a general anesthetic agent, which is a specific probe used for assessing the metabolic activity of the CYP3A4/1 enzymes and it is not a substrate for P-gp (Takano et al., 1998; Fromm, 2004; Cécile et al., 2007).

## Materials and Methods

### Materials

Losartan, verapamil, and ritonavir pure substances were obtained as gift samples from Dr Reddy’s Laboratories, Hyderabad, India. Cyclosporine A was obtained as gift sample from Bharat Serums limited, Mumbai, India. Midazolam and ketoconazole pure substances were obtained as gift samples from Lupin Laboratories, Pune, India. All the solvents were of HPLC grade obtained from Rankem India. Potassium dihydrogen orthophosphate, orthophosphoric acid was purchased from E. Merck Limited, Mumbai, India and all other chemicals were obtained from Hi Media, Mumbai, India.

### Composition of Perfusion Solutions

The perfusion buffer composition was as follows: CaCl_2_ × 2H_2_O, 0.98 mM, KCl 2.58 mM, Na_2_HPO4 0.66 mM, NaH_2_PO4 5.1 mM, NaCl 84 mM, D-glucose 3.0 mM with pH 6.8 (with NaOH) phenol red (50 mg L^-^1) was added to the solution as a non-absorbable marker. The pH was adjusted to 7.4 and the osmolality, measured by the freezing point depression method, was 230 ± 10 mOsm kg^-^1 (Osmette A, Precision Systems Inc., Natick, MA, USA) isotonic for amphibian (Franco et al., 2008). Preliminary experiments showed that there was no adsorption of the compounds to the catheters and the tubing. Test drug concentrations used in the perfusion studies were selected by dividing the maximum prescribed dose of the drug by 250 ml, the accepted gastric volume, so as to represent maximal concentration in the intestinal segment. Solutions of probe drugs were prepared with blank perfusion buffer. Probe drugs and inhibitors used in the study, if insoluble in perfusion buffer were solubilized using less than 1% methanol.

### Analytical Methods

Samples were analyzed by reverse-phase HPLC. A liquid chromatographic system (HPLC; Agilent) with a solvent pump (Agilent 1100 binary pump) and UV-vis multiple wavelength detector (MWD-G1365B, was used. Chemstation^®^software was used for data acquisition, reporting, and analysis. The C-18 reverse phase column used for chromatographic separation was Zorbax (particle size 5 μ, pore size 10 nm, dimension 4.6 mm × 250 mm) of Agilent technologies. Mass spectral analysis of the perfusates, wherever required was done by Agilent LC-MSD ion trap in positive ion mode.

### Chromatographic Conditions

For losartan, the mobile phase was a mixture of 50% acetonitrile, 50% 0.05 M Phosphate buffer (Potassium dihydrogen phosphate, (pH adjusted to 3.2 with ortho phosphoric acid) and 0.5% triethylamine. For midazolam the mobile phase was a mixture of 0.05 M phosphate buffer (pH adjusted to 3.5 with orthophosphoric acid) and acetonitrile (65:35). The mobile phase was filtered through sintered glass filter P5 (1–1.6 μm; Winteg, Germany) and degassed in sonicator (Liarre, Italy). The mobile phase was pumped in isocratic mode at a flow rate of 1ml/min at ambient temperature for both the drugs. The UV detection was accomplished at 250 nm for losartan and 254 nm for midazolam. Quantification of the compounds was carried out by measuring the peak areas in relation to those of standards chromatographed under the same conditions.

### Frog *In-Situ* SPIP Technique

Frog *in situ* perfusion studies were performed using established SPIP method for rats (Varma and Panchagnula, 2005) with few modifications. Animal care and handling throughout the experimental procedure were performed in accordance with the “Principles of Laboratory Animal Care” (NIH publication #85–23, revised in 1985). Frogs of the species Rana tigrina, were used for the experiments. Frogs were fasted for 24 h prior to the start of the experiment. Each frog was anesthetized and maintained with a combination of intra-peritoneal injection of 230 mg/kg of phenobarbitone sodium and 25 mg/kg of thiopentone sodium. After the onset of deep anesthesia, abdomen was opened by a midline longitudinal incision, and approximately 15–20 cm length of intestine immediately after stomach was selected, rinsed with frog’s ringer and cannulated on both sides. Care was taken in handling the small intestine to minimize the blood loss. Initially the contents of the intestine were flushed out with blank perfusion solution, then with the test solution, and then perfused with test solutions at a flow rate of 0.2 ml/min using syringe pump for 90 min after 30 min of equilibration. The perfusate samples were collected at every 10 min. Water flux was quantified with the help of concentration change of phenol red (non-absorbable inert marker). The length and radius of the perfused segment was measured at the end of the experiment and the animal was euthanized by quick removal of the heart. Permeability for each drug with or without inhibitor at different concentrations was determined in six frogs and the results were presented as mean ± SD. Samples were stored at -20°C until analysis. Effective permeability coefficient (*P*_eff_) of Losartan and Midazolam at concentrations 20, 40 mM, respectively, were calculated alone and also in presence of inhibitors of P-gp and/or CYP3A4 such as cyclosporine, verapamil, ketoconazole, ritonavir, and also combination of cyclosporine and ketoconazole each at increasing concentrations 1, 10, 30, 50 μM, respectively.

## Data Analysis

### Effective Permeability Coefficient (*P*_eff_)

Effective permeability coefficient (*P*_eff_) was calculated from the steady-state concentration of compounds in the collected perfusate (Komiya et al., 1980). Steady state, which was assessed by a constant concentration of phenol red, was reached 30 ± 40 min after the beginning of the experiment. *P*_eff_-value was calculated using equation (1), according to the parallel tube model (Sinko et al., 1991).

(1) $$P_{\text{eff}}=-\text{Q ln }[{\text{C}}_{\text{out corr}}{\text{/C}}_{\text{in}}]\text{/2πrl }\;$$

Where Q is perfusion flow rate (ml/min). C_in_ is inlet concentration (μg/ml). C_out(corr)_ is outlet concentration of compound which is corrected for water flux using phenol red concentration (μg/ml) [C_out(corr)__=_ C_outmeasured_ × (phenol red) in/(phenol red) out], *r* is the radius of the frog intestine (cm). One is the length of the intestinal segment (cm). The concentrations obtained from the perfusate were corrected for changes in the water flux at each time interval using the above equation.

## Results and Discussion

The effective permeabilities (*P*_eff_) were determined in frogs, *in situ*, from steady state values of C*_out_*_corr_/C_in_, for losartan, and midazolam. Steady state was attained in about 30–40 min, after beginning of the perfusion. Compounds were perfused alone and in presence of inhibitors to elucidate the role of P-gp and CYP3A4. Equilibrium was reached within 30–40 min, as assessed by the concentration of phenol red in the outlet. The recovery of phenol red was 98.3 ± 1.0%, in agreement with previous reports (Miller et al., 1987), indicating that the intestinal mucosa was not altered during the procedure. Effective permeability coefficient (*P*_eff_) of losartan and midazolam were calculated alone and also in the presence of cyclosporine and ritonavir which are well known inhibitors of P-gp and CYP3A4 (Benet et al., 2004). Cyclosporine and ritonavir were selected since they are dual inhibitors and the change in *P*_eff_ of losartan in presence of these inhibitors helps us to conclude the expression of P-gp and CYP3A4 in frog intestine. The effective permeability coefficient (*P*_eff_) values were also determined at steady state in the presence of ketoconazole, a CYP3A4 inhibitor, and verapamil, inhibitor of P-gp. Ketoconazole was selected since it is a potent inhibitor of CYP3A4 enzyme (Takahiro et al., 2002). Verapamil was selected since it inhibits only P-gp but not CYP3A4 (Verschraagen et al., 1999). Each inhibitor was tested for its inhibitory potential at four different concentrations to authenticate the results. Furthermore cyclosporine being a potent inhibitor of P-gp and ketoconazole, a potent inhibitor of CYP3A4 were used in combination at four different concentrations to determine their effect on intestinal permeability in frog.

**FIGURE 1:**
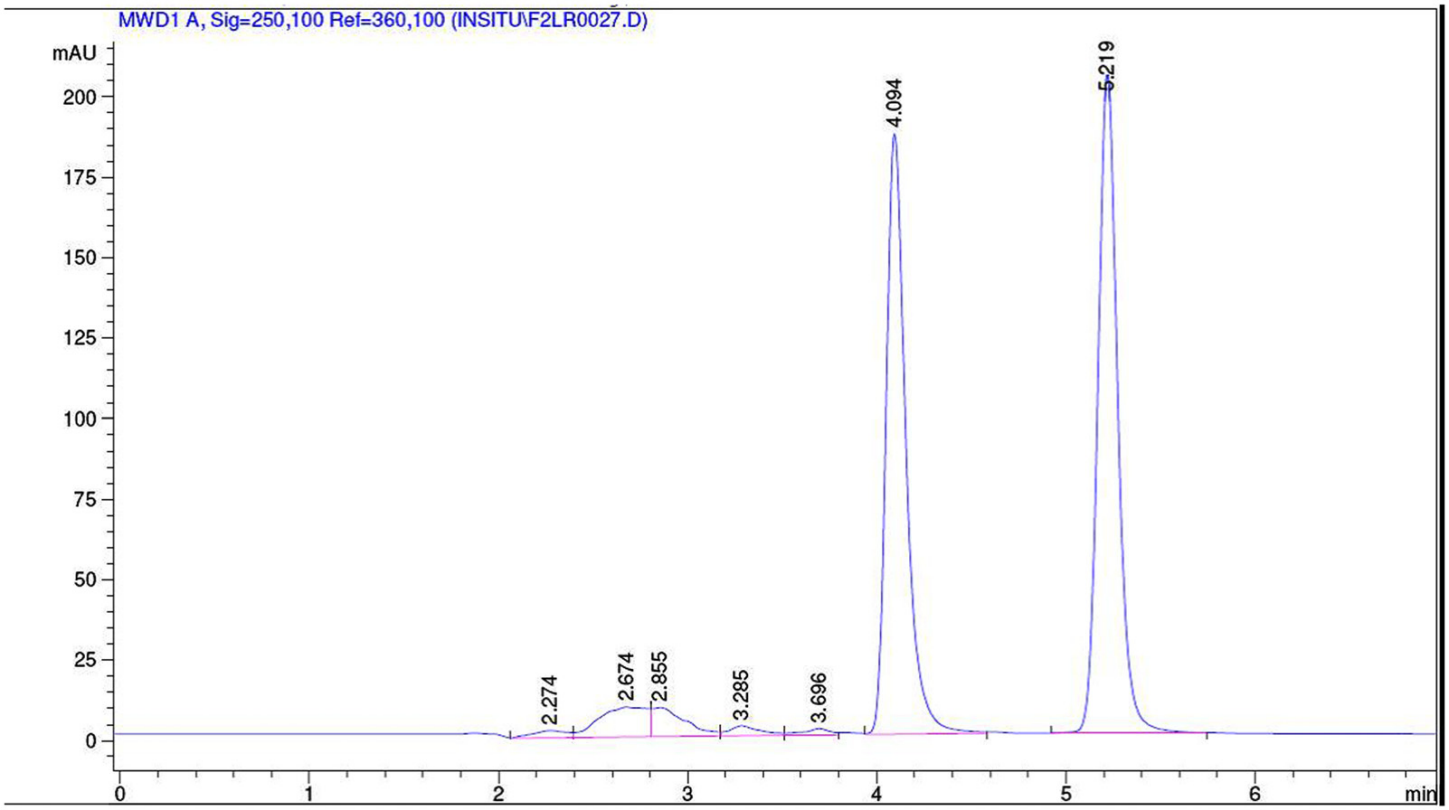
**Representative chromatogram of a sample from intestinal perfusion containing losartan and phenol red**.

**FIGURE 2:**
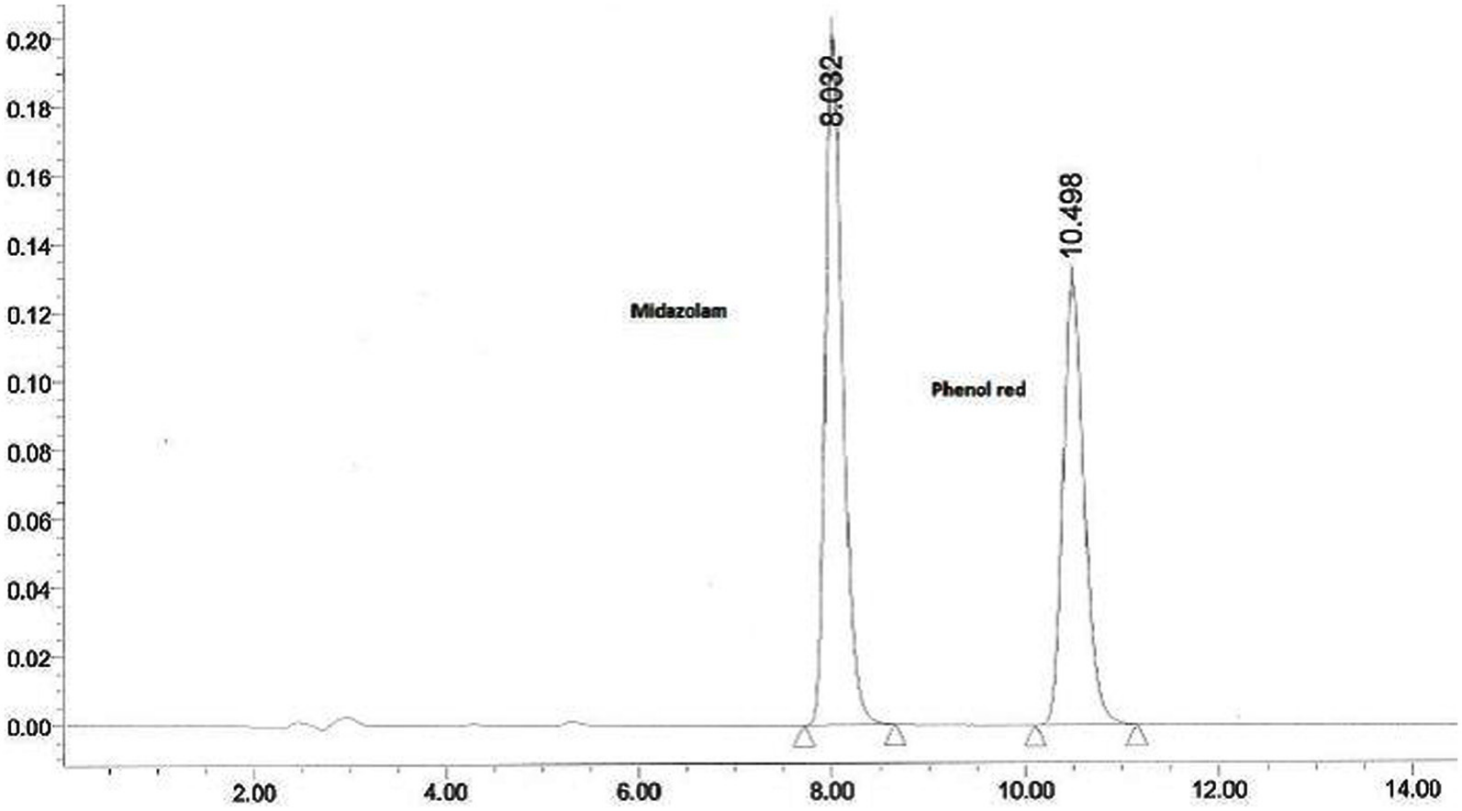
**Representative chromatogram of a sample from intestinal perfusion containing midazolam and phenol red**.

The collected outlet perfusate samples were stored at -20°C until analysis and they were analyzed by established RPHPLC methods from literature after optimization. In **Figures 1** and **2** the representative chromatograms of phenol red, losartan, and phenol red, midazolam are presented. There was no interference from the inhibitors. The retention times were 4.0 min and 5.2 min for phenol red and losartan, respectively, and for midazolam and phenol red they were 8.03 and 10.49min, respectively. Linearity was found by the six-point calibration curves for phenol red and losartan prepared in the range of 10–150 μg/ml. This concentration range was selected based on drug concentration used in permeability studies.

**FIGURE 3:**
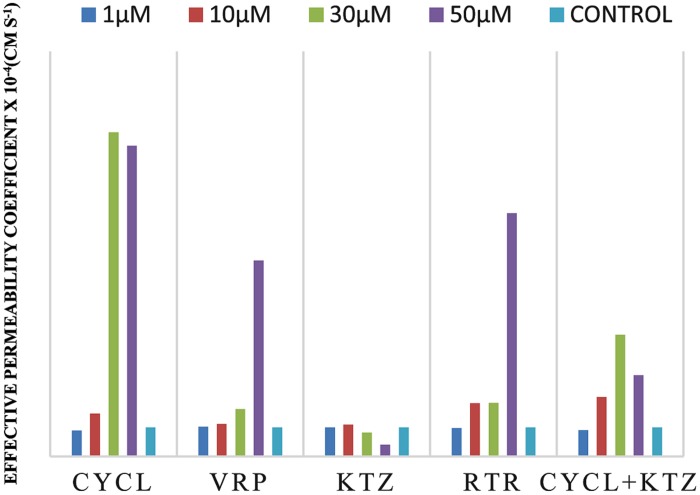
**Effective permeability coefficient of losartan in presence of Cyclosporine, Verapamil, Ketoconazole, Ritonavir, and also combination of Cyclosporine and ketoconazole at 1, 10, 30, 50 μM, respectively**.

**FIGURE 4:**
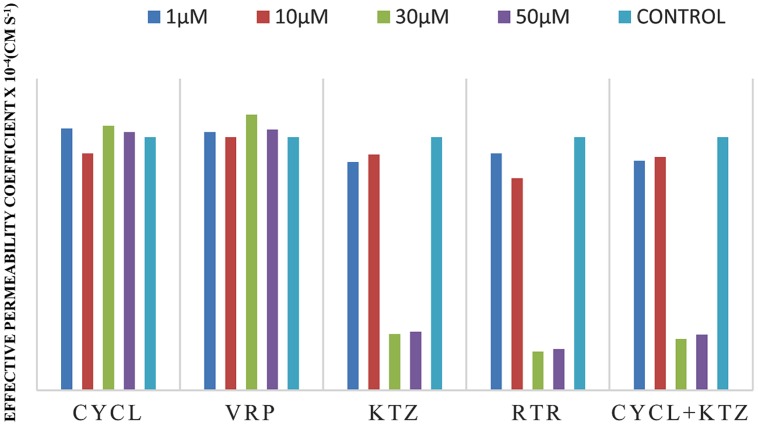
**Effective permeability coefficient of midazolam in presence of Cyclosporine, Verapamil, Ketoconazole, Ritonavir, and also combination of Cyclosporine and ketoconazole at 1, 10, 30, 50 μM, respectively**.

Effective permeability coefficient of losartan was found to be 0.427 ± 0.27 × 10^-4^cm/s when perfused alone and when it was co-perfused with inhibitors, significant change in *P*_eff_ was observed. Similarly, effective permeability coefficient of midazolam was found to be 2.03 ± 0.07 × 10^-4^cm/s when perfused alone and when it was co-perfused with inhibitors, no significant change in *P*_eff_ was observed. Comparison of effective permeability coefficient of losartan and midazolam in frog and other permeability models reported in literature is given in **Table 1**. Comparison of effective permeability coefficient of losartan and midazolam in frog in presence of cyclosporine, ritonavir which are very well known inhibitors of P-gp and CYP3A4, ketoconazole, a CYP3A4 inhibitor, and verapamil, inhibitor of P-gp at four concentrations (1, 10, 30, 50 μM, respectively) and also cyclosporine and ketoconazole, in combination (1:1) at these concentrations is depicted in **Figures 3** and **4**. The data are shown in **Tables 2** and **3**. Significant increase in the effective permeability coefficient of losartan was observed in the presence of cyclosporine at 30, 50 μm. 11.16-fold increase in *P*_eff_ was observed at 30 μm concentration and 10.70-fold increases in *P*_eff_ was observed at 50 μm concentration. Significant increase (8.37-fold increase) in the effective permeability coefficient of losartan was observed in the presence of ritonavir at 50 μm concentration. Verapamil showed 6.74-fold increase in effective permeability coefficient at 50 μm. These values indicate that losartan is actively effluxed by P-gp in the frog intestine since effective permeability coefficient increased significantly in presence of known inhibitors of P-gp such as cyclosporine, ritonavir, verapamil. In presence of ketoconazole significant change in effective permeability coefficient was not observed but in presence of cyclosporine and ketoconazole, in combination (1:1) at 30, 50 μM, respectively, showed significant change in effective permeability coefficient. Comparison of these values with that in presence of cyclosporine alone indicate that losartan is also metabolized probably by CYP3A4 enzymes or other isoforms of CYP enzymes.

**Table 1:** Effective permeability coefficient (10^-4^cm s^-1^) of losartan and Midazolam.

	Frog	Human	Rat	Caco-2
Losartan	0.427 ± 0.27	1.15^a^	*	0.022^b^
Midazolam	2.03 ± 0.07	*	2.08 ± 0.50^c^	*

*Values are mean ± SD of six values.*

*^a^Lennernäs, 2007.*

*^b^Andrea et al., 2000.*

*^c^Cécile et al., 2007.*

**Table 2:** Effective permeability coefficient (10^-4^cm s^-1^) of losartan in presence of inhibitors.

Inhibitors	1 μM	10 μM	30 μM	50 μM
Cyclosporine	0.38 ± 0.07	0.63 ± 0.12	4.80 ± 0.13	4.60 ± 0.04
Verapamil	0.44 ± 0.09	0.48 ± 0.02	0.70 ± 0.16	2.90 ± 0.14
Ketoconazole	0.43 ± 0.04	0.47 ± 0.13	0.35 ± 0.14	0.17 ± 0.03
Ritonavir	0.42 ± 0.05	0.79 ± 0.09	0.79 ± 0.17	3.60 ± 0.14
Cyclosporine + Ketoconazole	0.39 ± 0.02	0.88 ± 0.15	1.80 ± 0.18	1.20 ± 0.16

*Values are mean ± SD of six values.

**Table 3:** Effective permeability coefficient (10^-4^cm s^-1^) of Midazolam in presence of inhibitors.

Inhibitors	1 μM	10 μM	30 μM	50 μM
Cyclosporine	2.1 ± 0.06	1.9 ± 0.05	2.12 ± 0.14	2.07 ± 0.03
Verapamil	2.07 ± 0.12	2.03 ± 0.06	2.21 ± 0.05	2.09 ± 0.05
Ketoconazole	1.83 ± 0.11	1.89 ± 0.02	0.45 ± 0.08	0.47 ± 0.05
Ritonavir	1.90 ± 0.14	1.70 ± 0.09	0.31 ± 0.02	0.33 ± 0.08
Cyclosporine + Ketoconazole	1.84 ± 0.13	1.87 ± 0.03	0.41 ± 0.04	0.45 ± 0.03

*Values are mean ± SD of six values.

**FIGURE 5:**
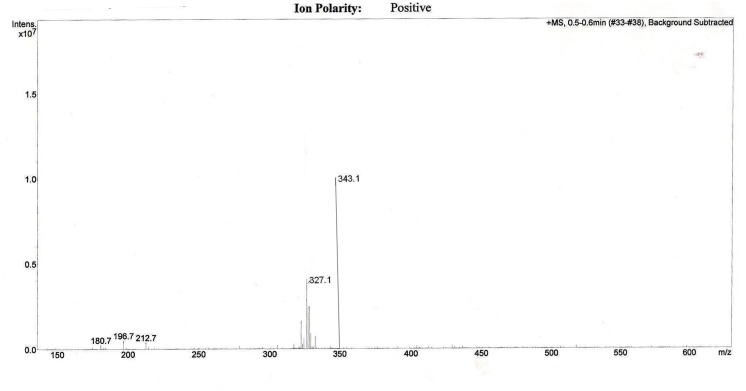
**Representative mass spectrum of a sample from intestinal perfusion containing peak corresponding to hydroxy midazolam**.

The effective permeability coefficient (*P*_eff_) of midazolam when perfused alone was found to be 2.03 ± 0.07 × 10^-4^cm/s. Significant change in effective permeability coefficient was not observed as expected in presence of any of the inhibitors of P-gp as it is well known that midazolam is not a substrate for P-gp. Moderate change in effective permeability coefficient was observed in presence of ketoconazole, a potent CYP3A4 inhibitor. This suggests that enzymes similar to CYP might have been expressed in frog intestine. Moreover in a study several new *CYP1* genes were identified in vertebrates (chicken, *Gallus gallus*, and frog, *Xenopus tropicalis*; Goldstone et al., 2007). In another study it was demonstrated that it is possible to determine the *P*_eff_-value for compounds which are substrates of CYP3A4 using *in situ* frog intestinal perfusion technique (Yerasi et al., 2010). To support this argument further, the perfusate samples were analyzed by mass spectroscopy. Mass spectra of some of the perfusate sample showed (M+H) peak, m/z 342 corresponding to hydroxymidazolam exhibiting mass difference of M+17 against the parent, the mass spectrum is shown in **Figure 5**. Comparison of P_eff_ calculated in frog with that of other available models and also humans suggested that the *P*_eff_-values are comparable and reflected well with human intestinal permeability. The *P*_eff_ of midazolam calculated in rat everted gut sac model and frog was found to be almost the same which is given in **Table 1**.

Comparision of the effect of inhibitors on the observed effective permeability coefficient (*P*_eff_) of probe drugs requires the understanding of physical model taken in to account to calculate effective permeability coefficient. Of the two frequently used mathematical models (Well stirred model, Parallel tube model) to calculate P_eff_, as stated earlier we have used parallel tube model since it takes in to account water absorption and secretion in to luminal drug solution in the intestinal segment. Considering it as a cylindrical tube, *P_eff_* calculated is inversely proportional to Cref lumen which is approximated as the logarithmic mean concentration (C) in the intestine [i.e., <C> = (*C*out -*C*in)/ln (*C*out/*C*in)]. P-gp expressed on the brush border membrane of enterocytes excretes its substrates into the lumen thus increasing the substrate’s *C*ref lumen. So when P-gp is inhibited *C*ref lumen decreases and *P*_eff_ increases, which are seen in case of probe drug losartan in presence of P-gp inhibitors such as cyclosporine and verapamil. CYP3A4 present in the gut wall metabolizes the substrate drug thus decreasing the *C*ref lumen. So when CYP3A4 is inhibited *C*ref lumen increases and *P*_eff_ decreases, which is seen in case of probe drug midazolam in presence of CYP3A4 inhibitors such as ketoconazole and ritonavir.

These results authenticate that P-gp and CYP3A enzymes are expressed in frog intestine and hence frog intestinal model can be reliably used as an animal model for studying intestinal permeability of drugs especially drugs which are substrates for P-gp and CYP enzymes. Further, comparison of the *P*_eff_-value of losartan obtained with that of reported human’s *P*_eff_ and Caco-2 cell data, and comparison of the *P*_eff_-value of midazolam with that of reported rat’s *P*_eff_, we can conclude that SPIP using frog as animal model can be reliably used in preclinical studies for permeability estimation. This model serves as a valuable alternative for permeability estimation of drugs which are dual substrates of P-gp and CYP3A4 since use of rat model and Caco-2 cell line model for such drugs are associated with limitations, though they are gold standards for permeability estimation of passively absorbed drugs such as model being static, absence of a mucus layer due to lack of mucous secreting cells, with different or no expression of metabolic enzymes (e.g., absence of CYPs), “Tighter” monolayer compared to human small intestine, wide Inter- and intra-laboratory variability of permeability data, Long differentiation period, Low expression of efflux transporters. Limitations of rat model include different expression profiles of transporters and metabolizing enzymes when compared with humans and the model is not suitable for high throughput screening. The frog model reported here appears to be promising for permeability assessment of drugs which are substrates for efflux proteins and CYP3A enzymes.

## Conclusion

Frog SPIP proves to be a promising model to calculate permeability coefficient of drugs which are substrates of P-gp and/or CYP3A4. Since this model mimics *in vivo* situation, most of the disadvantages of *in vitro* models are negated. It serves as a cost effective alternative to existing *in situ* models.

## Conflict of Interest Statement

The authors declare that the research was conducted in the absence of any commercial or financial relationships that could be construed as a potential conflict of interest.
